# Real‐world outcomes of FOLFIRINOX vs gemcitabine and nab‐paclitaxel in advanced pancreatic cancer: A population‐based propensity score‐weighted analysis

**DOI:** 10.1002/cam4.2705

**Published:** 2019-11-13

**Authors:** Kelvin K. W. Chan, Helen Guo, Sierra Cheng, Jaclyn M. Beca, Ruby Redmond‐Misner, Wanrudee Isaranuwatchai, Lucy Qiao, Craig Earle, Scott R. Berry, James J. Biagi, Stephen Welch, Brandon M. Meyers, Nicole Mittmann, Natalie Coburn, Jessica Arias, Deborah Schwartz, Wei F. Dai, Scott Gavura, Robin McLeod, Erin D. Kennedy

**Affiliations:** ^1^ Cancer Care Ontario Toronto ON Canada; ^2^ Sunnybrook Odette Cancer Centre University of Toronto Toronto ON Canada; ^3^ Canadian Centre for Applied Research in Cancer Control Toronto ON Canada; ^4^ Pharmacoeconomics Research Unit Cancer Care Ontario Toronto ON Canada; ^5^ Ontario Institute for Cancer Research Toronto ON Canada; ^6^ Cancer Centre of Southeastern Ontario Kingston ON Canada; ^7^ London Regional Cancer Program London ON Canada; ^8^ Hamilton Health Sciences Centre Hamilton ON Canada; ^9^ Mount Sinai Hospital Toronto ON Canada

**Keywords:** FOLFIRINOX, gemcitabine, outcomes, pancreatic cancer, real‐world evidence

## Abstract

**Background:**

In Ontario, FOLFIRINOX (FFX) and gemcitabine + nab‐paclitaxel (GnP) have been publicly funded for first‐line unresectable locally advanced pancreatic cancer (uLAPC) or metastatic pancreatic cancer (mPC) since April 2015. We examined the real‐world effectiveness and safety of FFX vs GnP for advanced pancreatic cancer, and in uLAPC and mPC.

**Methods:**

Patients receiving first‐line FFX or GnP from April 2015 to March 2017 were identified in the New Drug Funding Program database. Baseline characteristics and outcomes were obtained through the Ontario Cancer Registry and other population‐based databases. Overall survival (OS) was assessed using Kaplan‐Meier and weighted Cox proportional hazard models, weighted by the inverse propensity score adjusting for baseline characteristics. Weighted odds ratio (OR) for hospitalization and emergency department visits (EDV) were estimated from weighted logistic regression models.

**Results:**

For 1130 patients (632 FFX, 498 GnP), crude median OS was 9.6 and 6.1 months for FFX and GnP, respectively. Weighted OS was improved for FFX vs GnP (HR = 0.77, 0.70‐0.85). Less frequent EDV and hospitalization were observed in FFX (EDV: 67.8%; Hospitalization: 49.2%) than GnP (EDV: 77.7%; Hospitalization: 59.3%). More frequent febrile neutropenia‐related hospitalization was observed in FFX (5.8%) than GnP (3.3%). Risk of EDV and hospitalization were significantly lower for FFX vs GnP (EDV: OR = 0.68, *P* = .0001; Hospitalization: OR = 0.76, *P* = .002), whereas the risk of febrile neutropenia‐related hospitalization was significantly higher (OR = 2.12, *P* = .001). Outcomes for uLAPC and mPC were similar.

**Conclusion:**

In the real world, FFX had longer OS, less frequent all‐cause EDV and all‐cause hospitalization, but more febrile neutropenia‐related hospitalization compared to GnP.

## Graphical abstract

In the real world, implementation of universal public funding of FOLFIRINOX for metastatic pancreatic cancer was associated with improved overall survival, less frequent all‐cause emergency department visits, less frequent all‐cause hospitalization, but increased febrile neutropenia‐related hospitalization compared to patients treated with gemcitabine + nab‐paclitaxel. Expanding funding to include unresectable locally advanced pancreatic cancer was associated with a similar trend in benefits, but with improved absolute survival.

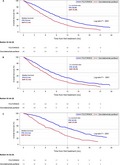

## 1 INTRODUCTION

Pancreatic cancer is the fourth leading cause of cancer‐related death in the USA as well as in Europe, with over 55 000 estimated new cases and over 44 000 estimated deaths in the United States in 2018.^1^, ^2^ Pancreatic cancer is projected to become the second leading cause of cancer‐related death by 2030.^3^ Despite some advances in the management of pancreatic cancer, outcomes for patients with this disease remain poor, with a 5‐year survival rate of 8% for all disease stages.^1^ The majority of new cases are diagnosed at distant stage (52%). For those, the 5‐year survival rate is only 3%. Only 10% of new diagnoses are local disease (32% 5‐year survival rate) and 29% are regional disease (12% 5‐year survival rate).^1^

For patients with advanced disease, FOLFIRINOX (fluorouracil, folinic acid, irinotecan, oxaliplatin) (FFX) or gemcitabine plus nanoparticle albumin‐bound paclitaxel (nab‐paclitaxel) (GnP) have emerged as possible treatment options.

The phase III MPACT trial compared GnP to gemcitabine monotherapy in patients with previously untreated metastatic adenocarcinoma of the pancreas (mPC), age ≥18 years, and Karnofsky performance status (KPS) 70 or greater.^4^, ^5^ Statistically significant improvements in overall survival (OS) (1‐year OS: 35% vs 22%, *P* < .001; 2‐year OS: 9% vs 4%, *P* = .01; median OS: 8.7 vs 6.6 months; OS HR 0.72 (95% CI 0.620‐0.825, *P* < .0001)) were observed, as were improvements in progression‐free survival (PFS) and overall response rate. Incidence of grade ≥3 treatment‐emergent adverse events (AEs) and all grade treatment‐emergent AEs was greater in patients receiving GnP, with a 3% rate of febrile neutropenia in the GnP group.^4^, ^5^

The PRODIGE/ACCORD 11 trial compared FFX against gemcitabine in patients with mPC and Eastern Cooperative Oncology Group performance status (ECOG PS).^6^ Median OS was improved (11.1 vs 6.8 months, HR 0.72 (95% CI 0.62‐0.83)) in FFX patients, as was PFS and objective response rate. The ACCORD 11 trial prospectively evaluated quality of life (QoL) and demonstrated that although QoL deteriorated in both treatment arms, patients who received FFX had better QoL than gemcitabine.^7^ However, FFX did have increased toxicity compared to gemcitabine, with 5.4% of patients having febrile neutropenia, and one population‐based study determined that only 26% of patients would have been found to meet the criteria to receive FFX treatment from the ACCORD 11 trial.^6^, ^8^

For mPC, the American Society of Clinical Oncology (ACSO) recommends FFX for patients with an Eastern Cooperative Oncology Group performance status (ECOG PS) of 0 to 1, favorable comorbidity profile, patient preference, and a support system for aggressive medical therapy, and access to chemotherapy port and infusion pump management services.^9^, ^10^ ASCO recommends GnP for patients of a ECOG PS 0 to 1, relatively favorable comorbidity profile, and a patient preference and support system for relatively aggressive medical therapy. Gemcitabine monotherapy is recommended for patients with an ECOG PS of 2 or patients with worse comorbidity profiles. Neither the MPACT nor the PRODIGE/ACCORD 11 trial included patients with unresectable, locally advanced pancreatic cancer (uLAPC), but rather only mPC. As such, ASCO recommends a combination regimen for first‐line therapy for uLAPC based on extrapolation from the MPACT and PRODIGE/ACCORD 11 trials, but cannot recommend one regimen over another due to lack of clear evidence.^11^

Although the pivotal trials of both FFX and GnP have shown both treatments improve OS, PFS as well as response rate, distinctive patient populations, and differences in trial design limit the generalizability of indirect comparisons**.** Furthermore, the ACCORD trial was conducted only in France, whereas the MPACT trial involved centers internationally. Direct comparisons of FFX and GnP are not expected. Results from indirect comparisons, however, have been conflicting. In one Bayesian meta‐analysis, a trend toward improvement in OS for FFX was found compared to GnP probabilistically, with no obvious difference in toxicities.^12^ In a different indirect comparison study, FFX again appeared to improve OS compared to GnP, however, with significantly worse neutropenia for FFX and significantly worse fatigue for GnP.^13^ Real‐world evidence may be able to address these inconsistencies as well as bridge the evidence gap between trial data and real‐world patients regarding both the lack of a direct comparison of FFX vs GnP, as well as the lack of studies evaluating uLAPC. Thus, this study aimed to examine the real‐world comparative effectiveness and safety of publicly funded FFX vs GnP in Ontario, Canada for patients with advanced pancreatic cancer, and specifically, patients with uLAPC and patients with mPC.

## 2 METHODS

### 2.1 Funded treatment options

Ontario publicly funded FFX as a first‐line treatment for mPC as of November 7, 2011 and for uLAPC as of April 17, 2015. GnP as a first‐line treatment for patients with mPC or uLAPC was publicly funded as of April 17, 2015. Second‐line oxaliplatin and nano‐liposomal irinotecan were not funded in Ontario during our study period.

### 2.2 Study population

The study protocol was approved by the institutional ethics committee of Sunnybrook Health Sciences Centre. Patients who received FFX or GnP as first‐line treatment were identified from the New Drug Funding program (NDFP) database. Patients whose first chemotherapy treatment occurred between April 17, 2015 (the start date of universal public funding of GnP in Ontario) to March 31, 2017 were included in the study. Patients who received FFX as first‐line treatment for mPC before April 17, 2015 were excluded to create a more comparable contemporary cohort to compare FFX vs GnP.

Baseline characteristics and outcomes were obtained through data linkage of patient's receiving FFX or GnP to the Ontario Cancer Registry (OCR), Cancer Activity Level Reporting (ALR), Discharge Abstracts Database (DAD), the National Ambulatory Care Reporting System (NACRS), and the Registered Persons Database (RPDB) by patients’ unique health card number. Canadian 2016 Census data were used to obtain patients’ rural/urban status and neighborhood income quintiles based on postal code of patients’ residence. Details see Appendix Data sources and Appendix Figure S1.

### 2.3 Outcomes

Our primary effectiveness outcome was OS, defined as the time from date of first‐line treatment to death or end of follow‐up if censored. Patients were followed up to Aug. 31, 2017. Our safety outcomes included all‐cause hospitalization, all‐cause emergency department (ED) visits and hospitalizations for febrile neutropenia during the treatment period.^14^ Treatment period was defined as the period from date of first treatment to date of last treatment plus 30 days or death, whichever occurred first. Common diagnoses of hospitalization were also collected. Patients continuing first‐line treatment after July 31, 2017 were excluded from the safety analysis, retaining only patients who stopped treatment on or before July 31, 2017 to ensure all patients had a minimum follow‐up window of 30 days after the last dose of first‐line treatment to examine all‐cause hospitalizations, all‐cause ED visits, and hospitalizations for febrile neutropenia.^15^ Febrile neutropenia was defined using neutropenia as most responsible diagnosis code for hospitalization or with fever or infection as most responsible diagnosis code plus neutropenia diagnosis code.

### 2.4 Statistical analysis

Descriptive statistics were used to summarize baseline characteristics. Age was calculated at patients’ first treatment. Patients’ postal code, ECOG PS, and mPC indicator were obtained when patients were enrolled to receive treatment. Previous adjuvant gemcitabine and previous radiation treatment were defined as any adjuvant gemcitabine or radiation prior to patients’ first treatment on FFX or GnP. Previous pancreatic resection and Charlson comorbidity index were calculated using DAD data looking back 2 years from patients’ first treatment.

Propensity score analysis using inverse probability of treatment weighting (IPTW) was conducted to account for potential confounders when comparing between FFX and GnP. The IPTW method can remove systematic differences between FFX and GnP on observed characteristics to a comparable degree compared to propensity score matching, without having to reduce the current sample size to estimate the average treatment effect.^16^ Propensity score was estimated using a logistic regression model with the treatment group, FFX or GnP, as the dependent variable, regressed on the potentially confounding variables: age, gender, previous adjuvant gemcitabine, previous radiation treatment, previous pancreatic resection, Charlson comorbidity index, ECOG PS, mPC, rural urban status, and income quintile. Weight was defined as the inverse propensity score of the treatment patients actually received. The weighted standardized difference was calculated for all the baseline characteristics to assess the balance of the baseline characteristics in the cohort after applying the weight. The rule of thumb, that a standard difference is less than 0.1, has been taken to indicate an adequate balance between treatment groups.^17^ Mean and standard deviation were calculated for continuous variables, whereas frequency and percentage were calculated for binary or categorical variables. Differences between baseline characteristics for FFX and GnP patients were tested by *t*‐test for continuous variables and chi‐square test for binary or categorical variables.

OS was assessed using Kaplan‐Meier survival curves, log‐rank test, and Cox proportional hazard models weighted by the inverse propensity score. For safety outcomes, binary outcomes of all‐cause ED visit, all‐cause hospitalization, and hospitalization for febrile neutropenia during treatment periods were defined, and the weighted odds ratios for all‐cause hospitalization and ED visit during treatment periods were estimated from weighted (IPTW) logistic regression models.

All analyses were done in the main cohort and the mPC and uLAPC subcohorts, separately.

All two‐sided *P* values <.05 were considered statistically significant. SAS version 9.4 (SAS Institute, Cary, North Carolina) was used to conduct the analyses.

### 2.5 Sensitivity analysis

Two sensitivity analyses were performed to examine the robustness of our results. First, multivariable analyses were conducted adjusting the same potential confounders used in the calculation of propensity score. Multivariable proportional hazards model was adopted to estimate an adjusted hazard ratio for OS and multivariable logistic regression models were adopted to estimate the adjusted odds ratio of all‐cause hospitalization or all‐cause ED visits during the treatment period. Second, we counted the number of all‐cause hospitalizations, number of all‐cause ED visits, and hospitalizations for febrile neutropenia during the patients’ treatment periods, and adopted negative binomial models to estimate the rate ratio (RR) of all‐cause hospitalization, all‐cause ED visits and hospitalization for febrile neutropenia accounting for differences in patients’ treatment periods. Univariate analyses were conducted for proportional hazards model, logistic regression, and negative binomial models as well.

## 3 RESULTS

### 3.1 Study population

The data source and the creation of the study population are shown in Appendix Figures S1 and S2. From April 17, 2015 to March 31, 2017, there were 1146 patients who received FFX or GnP as the first‐line treatment of advanced pancreatic cancer. Sixteen patients were excluded due to missing data for the following variables: metastatic pancreatic indicator, ECOG PS, or rural/urban status. The final cohort included 1130 patients.

### 3.2 Baseline characteristics

Baseline characteristics of the study population by treatment group are shown in Table 1. Baseline characteristics for mPC and uLAPC subcohorts by treatment group are shown in Appendix Tables S1 and S2. Among 1130 patients with advanced pancreatic cancer, 55.93% received FFX as first‐line treatment. Mean age was 65.1 ± 9.6 years, and 42.92% were women. More patients had received previous adjuvant gemcitabine (12.34% FFX vs 7.03% GnP) in FFX group, whereas more patients had received previous radiation treatment (10.44% GnP vs 6.49% FFX) in the GnP group. More patients were diagnosed with mPC (76.31% vs 65.82%) and had higher ECOG PS (81.73% vs 59.65%) in the GnP group compared to patients who received FFX. There was no significant difference of previous pancreatic resection, Charlson comorbidity score, rurality or income quintile between FFX and GnP group.

**Table 1:** Baseline characteristics for advanced pancreatic cancer patients by chemotherapy treatment

Characteristics	Before IPTW			After IPTW		
	FOLFIRINOX (n = 632)	Gemcitabine/nab‐paclitaxel (n = 498)	*P*‐value	FOLFIRINOX (n = 632)	Gemcitabine/nab‐paclitaxel (n = 498)	Weighted standardized difference
Age at first treatment (mean ± SD)	61.83 ± 9.12	69.14 ± 8.69	<.0001	64.63 ± 12.06	64.35 ± 15.45	0.0201
Female	287 (45.41%)	198 (39.76%)	.0567	42.26%	42.27%	0.0002
Previous adjuvant gemcitabine	78 (12.34%)	35 (7.03%)	.0031	10.12%	8.60%	0.0523
Previous radiation	41 (6.49%)	52 (10.44%)	.0163	8.88%	8.52%	0.0129
Previous pancreatic resection	102 (16.14%)	64 (12.85%)	.1211	14.93%	15.65%	0.0202
Metastatic	416 (65.82%)	380 (76.31%)	.0001	70.45%	68.45%	0.0434
Charlson comorbidity index 1+	183 (28.96%)	162 (32.53%)	.1952	29.57%	30.84%	0.0277
ECOG PS 1+	377 (59.65%)	407 (81.73%)	<.0001	69.35%	67.57%	0.0383
Urban	548 (86.71%)	439 (88.15%)	.4686	13.05%	12.58%	0.0141
Income quintile						
1 (lowest)	80 (12.66%)	85 (17.07%)	.2172	14.25%	16.33%	0.0580
2	102 (16.14%)	82 (16.47%)		17.26%	14.95%	0.0627
3	111 (17.56%)	96 (19.28%)		17.77%	17.16%	0.0159
4	131 (20.73%)	98 (19.68%)		21.00%	20.87%	0.0032
5 (highest)	137 (21.68%)	92 (18.47%)		20.13%	20.99%	0.0213
unknown	71 (11.23%)	45 (9.04%)		9.60%	9.70%	0.0032

Abbreviations: ECOG PS, Eastern Co‐operative Oncology Group performance status; IPTW, Inverse probability treatment weighting; SD, standard deviation.

Weighted standardized difference on all baseline characteristics were calculated for the overall cohort (Table 1), mPC subcohort (Appendix Table S1), and uLAPC subcohort (Appendix Table S2). A standardized difference of less than 0.1 was considered to indicate a good balance of covariates between FFX and GnP group. All the standardized differences in our analysis were less than 0.1 except lowest income quintile in the uLAPC subcohort were 0.1294. We did a sensitivity analysis by adding the income quintile in the weighted cox regression and negative binomial models and found the results were quite robust (Appendix Table S4).

A total of 139 (12.3%) patients of 1130 had subsequent treatments. Of these patients, 129 (92.8%) had initial FFX and 10 (7.2%) had initial GnP. For patients who had initial FFX, 95 (73.6%) proceeded to subsequent gemcitabine and 34 (26.4%) proceeded to subsequent GnP. For patients who had initial GnP, nine (90%) proceeded to subsequent gemcitabine and one (10%) proceeded to subsequent FFX.

### 3.3 Overall survival

**Figure 1:**
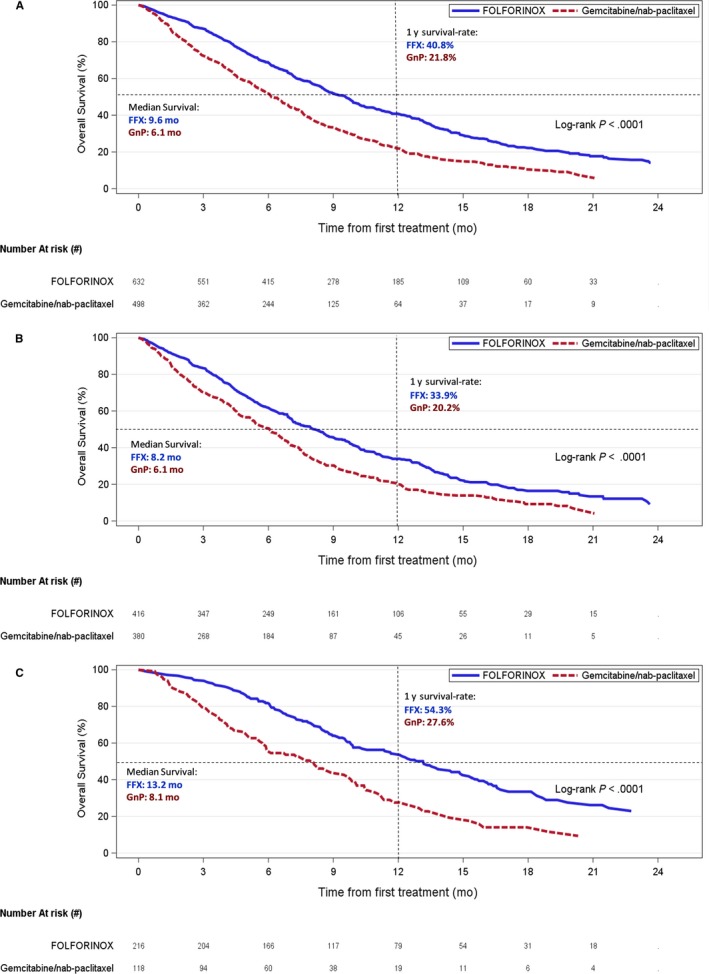
Overall survival for patients with (A) advanced pancreatic cancer, (B) metastatic pancreatic cancer (mPC), and (C) unresectable locally advanced pancreatic cancer (uLAPC). FFX, FOLFIRINOX; GnP, gemcitabine + nab‐paclitaxel

**Figure 2:**
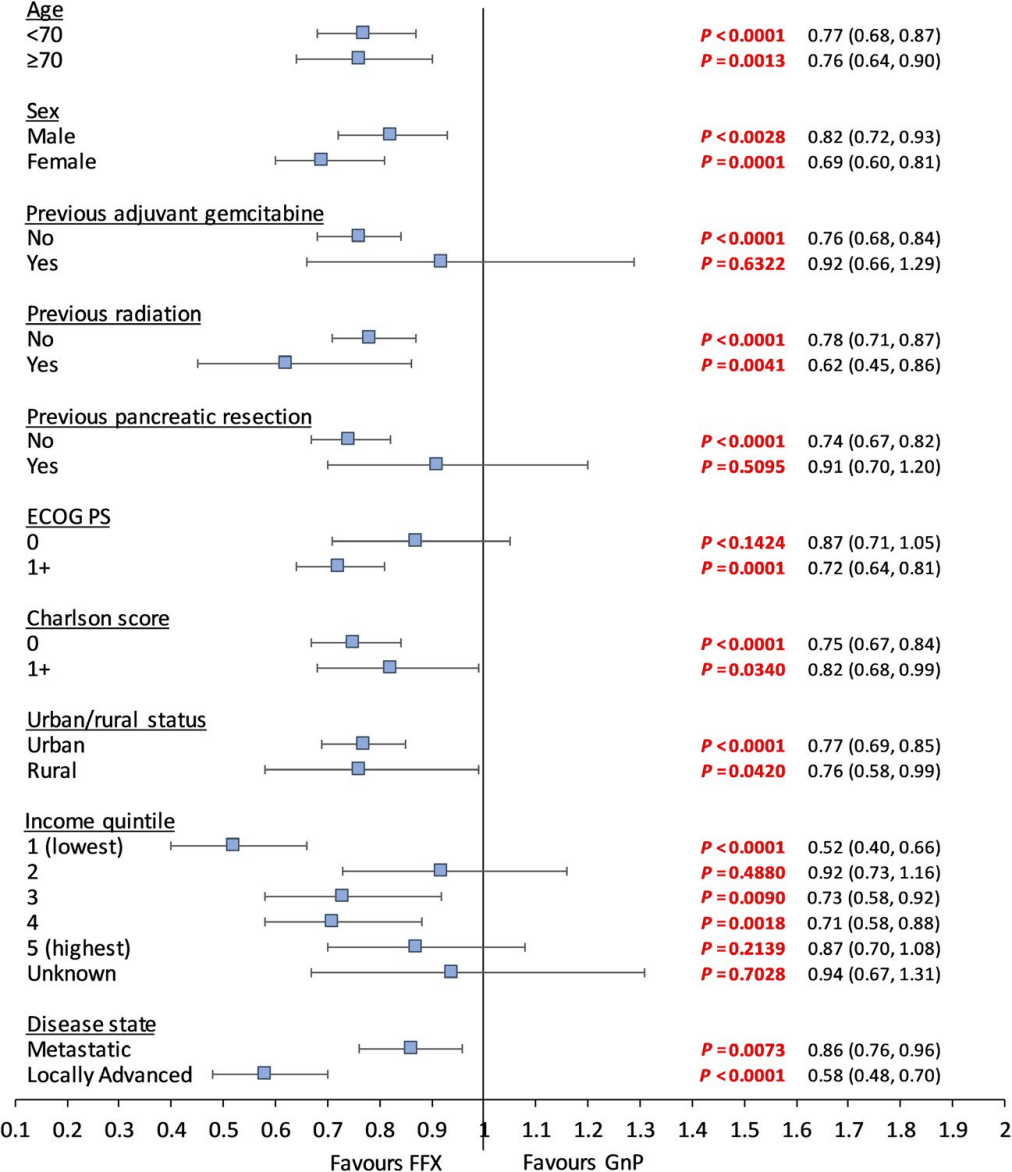
Forest plot of hazard ratios of overall mortality for FFX vs GnP in each sub‐group from weighted Cox proportional hazard model. ECOG PS, Eastern Co‐operative Oncology Group performance status; FFX, FOLFIRINOX; GnP, gemcitabine + nab‐paclitaxel

Patients were followed up until Aug. 31, 2017 with average follow‐up of 8 months and maximum follow‐up time of 28 months. Crude median survival was 9.6 and 6.1 months for patients who received FFX and GnP, respectively. In 334 patients with uLAPC, crude median survival was 13.2 and 8.1 months for FFX and GnP groups; in 796 patients with mPC, crude median survival was 8.2 and 6.1 months for FFX and GnP groups. Kaplan‐Meier curves for overall cohort, mPC and uLAPC subcohorts are shown in Figure 1A‐C. After 1 year, 40.8% patients survived in FFX group, whereas 21.8% survived in GnP group. After applying IPTW, standard difference for all baseline variables is less than 0.1 in the overall cohort (Table 1). OS was significantly improved for patients who received FFX compared to GnP (Table 2). Comparing FFX vs GnP, in the overall cohort, weighted HR (95% CI) was 0.77 (0.70, 0.85), and multivariable adjusted HR was 0.73 (0.62, 0.85). As well, OS was significantly improved in mPC and uLAPC subcohorts for FFX patients as compared to GnP patients. Weighted HR of overall mortality for each subgroup defined from the listed covariates in Table 1 were calculated (forest plot in Figure 2). The crude median survival for patients with initial FFX who received subsequent treatments was 10.4 months (9.7, 12.6). The crude median survival for patients with initial GnP who received subsequent treatments was 8.4 months (3.2, 12.2).

**Table 2:** Hazard ratios of overall mortality for FOLFIRINOX vs gemcitabine/nab‐paclitaxel

Patients		Crude Cox proportional hazard model	Weighted Cox proportional hazard model	Adjusted Cox proportional hazard model
All pancreatic cancer	HR (95%CI)	0.60 (0.53, 0.69)	0.77 (0.70, 0.85)	0.73 (0.62, 0.85)
	*P*‐value	<.0001	<.0001	<.0001
Metastatic pancreatic cancer	HR (95%CI)	0.68 (0.58, 0.80)	0.86 (0.77, 0.97)	0.78 (0.65, 0.94)
	*P*‐value	<.0001	0.0115	0.0077
Unresectable locally advanced pancreatic cancer	HR (95%CI)	0.50 (0.38, 0.67)	0.57 (0.47, 0.70)	0.58 (0.42, 0.79)
	*P*‐value	<.0001	<.0001	.0005

### 3.4 Safety outcomes

We excluded 104 patients who were continuing first‐line treatment after July 31, 2017. In the overall cohort, less frequent ED visits and hospitalizations were observed in FFX group (ED: 67.8%; Hospitalization: 49.2%) compared to GnP group (ED: 77.7%; Hospitalization: 59.3%). More frequent febrile neutropenia‐related hospitalization was observed in FFX (5.8%) compared to GnP patients (3.3%). After applying IPTW, risk of ED visit and hospitalization were significantly lower for FFX vs GnP group. The weighted OR (95% CI) for ED visit was 0.68 (0.56, 0.83), *P* = .0001; whereas for hospitalization was 0.76 (0.64, 0.91), *P* = .002. Risk of febrile neutropenia‐related hospitalization was significantly higher for FFX vs GnP group, with weighted OR (95% CI) of 2.12 (1.35, 3.31), *P* = .001.

Similarly, less frequent ED visits and hospitalization were observed in FFX (ED: 66.9%; Hospitalization: 49.9%) compared to GnP group (ED: 77.8%; Hospitalization: 58.2%) in the mPC subcohort. After applying IPTW, risk of ED visits was significantly lower for FFX vs GnP patients. The weighted OR (95% CI) for ED was 0.63 (0.50, 0.80), *P* = .0001, whereas the risk of febrile neutropenia‐related hospitalization was significantly higher for FFX vs GnP patients with weighted OR (95% CI) of 2.10 (1.22, 3.63), *P* = .008. There is no significant difference of risk of ED visit and hospitalization for febrile neutropenia between FFX and GnP group in the uLAPC subcohort, but the risk of all‐cause hospitalizations for FFX patients was significantly lower with weighted OR (95% CI) of 0.62 (0.45, 0.85), *P* = .003. Similar trends were observed from multivariable adjusted logistic regression (Table 3).

**Table 3:** Odds ratio (95% CI) of toxicity outcomes for FOLFIRINOX vs gemcitabine/nab‐paclitaxel

Patients	Outcome	Crude logistic regression	Weighted logistic regression	Adjusted logistic regression
All pancreatic cancer	All‐cause ED visit	0.61 (0.46, 0.80)	0.68 (0.56, 0.83)	0.66 (0.47, 0.91)
		*P* = .0005	*P* = .0001	*P* = .0116
	All‐cause hospitalization	0.66 (0.52, 0.85)	0.76 (0.64, 0.91)	0.70 (0.53, 0.94)
		*P* = .0013	*P* = .0022	*P* = .0158
	Hospitalization for febrile neutropenia	1.81 (0.97, 3.38)	2.12 (1.35, 3.31)	1.45 (0.72, 2.95)
		*P* = .0610	*P* = .0010	*P* = .3007
Metastatic pancreatic cancer	All‐cause ED visit	0.58 (0.41, 0.80)	0.63 (0.50, 0.80)	0.59 (0.40, 0.87)
		*P* = .0011	*P* = .0001	*P* = .0073
	All‐cause hospitalization	0.71 (0.53, 0.96)	0.86 (0.70, 1.06)	0.76 (0.54, 1.06)
		*P* = .0238	*P* = .1694	*P* = .1092
	Hospitalization for febrile neutropenia	1.65 (0.78, 3.53)	2.10 (1.22, 3.63)	1.65 (0.69, 3.93)
		*P* = .1925	*P* = .0078	*P* = .2594
Unresectable locally advanced pancreatic cancer	All‐cause ED visit	0.68 (0.39, 1.17)	0.90 (0.63, 1.29)	0.82 (0.45, 1.52)
		*P* = .1651	*P* = .5772	*P* = .5373
	All‐cause Hospitalization	0.54 (0.33, 0.88)	0.62 (0.45, 0.85)	0.57 (0.33, 0.99)
		*P* = .0141	*P* = .0032	*P* = .0471
	Hospitalization for febrile neutropenia	1.96 (0.63, 6.13)	1.78 (0.85, 3.74)	1.45 (0.41, 5.06)
		*P* = .2449	*P* = .1273	*P* = .5632

Abbreviations: CI, confidence interval; ED, emergency department.

The results of the rate ratio regression sensitivity analyses are congruent with the results of binominal logistical regressions with respect to ED visits and hospitalization. In the overall cohort, significantly less frequent ED visits, less all‐cause hospitalizations and more febrile neutropenia related hospitalizations were observed in patients who received FFX compared to GnP; weighted RR were 0.78 (0.68, 0.91), 0.71 (0.59, 0.85), and 2.65 (1.90, 3.68) for ED visits, all‐cause hospitalizations, and febrile neutropenia related hospitalizations, respectively. For details see Appendix Table S3.

In the advanced pancreatic cancer patients who received treatments, the common diagnosis of hospitalization during treatment included the following: malignant neoplasm of pancreas (16.84%), palliative care (7.95%), obstruction of bile duct (4.77%), acute pancreatitis (2.56%), neutropenia (2.39%), other and unspecified intestinal obstructions (1.97%), unspecified fever (1.87%), unspecified sepsis (1.80%), cholangitis (1.69%), pulmonary embolism (1.52%), mechanical complication of GI prosthetic devices, implants, and grafts (1.49%), and unspecified pneumonia (1.45%). These causes are consistent with symptoms and complications of pancreatic cancer and side‐effects of chemotherapy treatment.

## 4 DISCUSSION

In this large real‐world population‐based study of the most populous province in Canada where FFX and GnP are universally publicly funded for both metastatic and locally advanced pancreatic cancer, OS was better among patients who received first‐line FFX compared with GnP for advanced pancreatic cancer. This remained true for not only all advanced pancreatic cancer, but the mPC and uLAPC subcohorts. The adjusted risk of mortality decreased by 27% for patients who received FFX compared with GnP for all advanced pancreatic cancer patients. FFX appeared to lead to less frequent all‐cause ED visits and all‐cause hospitalization, but more hospitalizations for febrile neutropenia.

Compared to the PRODIGE/ACCORD 11 and MPACT trials which both included only mPC patients, median OS for mPC was lower for both FFX and GnP in our real‐world study (FFX real world: 8.2 months, PRODIGE/ACCORD 11:11.1 months; GnP real world: 6.1 months, MPACT: 8.7 months).^4^, ^6^ These results are concordant with others comparing randomized phase III trial outcomes to real‐world practice.^18^ Differences may also be due to differing study populations. Our real‐world analysis included a greater percentage of ECOG PS 1+ patients receiving GnP compared to MPACT (67.57% after IPTW vs approximately 42% in MPACT), although similar to PRODIGE/ACCORD 11 (62.5%). Prior treatments of chemotherapy, radiation, and pancreatic resection were all more common compared to MPACT (PRODIGE/ACCORD 11 excluded patients with previous chemotherapy or radiotherapy for measurable lesions).

In a recently published adjuvant trial, FFX significantly improved OS compared to gemcitabine (FFX: 54.4 months, gemcitabine: 35.0 months, difference of 19.4 months; HR 0.64, *P* = .003).^19^ In another adjuvant trial, GnP did not significantly improve the primary endpoint of disease‐free survival (DFS) compared to gemcitabine (HR 0.88, *P* = .1824).^20^ While no direct comparison between FFX and GnP has been conducted, our findings of FFX superiority over GnP are supported by the findings of these two adjuvant trials as well as the relative magnitude of benefits observed in the metastatic trials.

In our Ontario, Canada‐based study, a significant difference favoring FFX was observed in the mPC subcohort. The median OS observed in our study supports that reported in another Canadian real‐world study which included patients treated with palliative intent FFX, prior to the start of public funding for uLAPC.^21^ Contrasting our results, two real‐world studies in the United States have reported no significant differences in survival between FFX and GnP. One study reported medians similar to the MPACT and PRODIGE/ACCORD 11 trials, however, is limited by a small sample size.^22^ The other reported much longer real‐world medians—2.7 and 3.4 months longer than the PRODIGE/ACCORD 11 and MPACT trials, as well as 5.8 months and 6.0 months longer than our study for FFX and GnP, respectively—however, involved a highly selective patient population requiring voluntary participation of physicians who then reviewed randomly selected charts from two waves of patients.^23^ Compared to existing literature, the outcomes observed in our study represent that of a more real world, nonselective view of the entire publicly funded population.

In a single‐center study in South Korea, mPC patients receiving GnP actually demonstrated significantly longer OS than those receiving FFX at 11.4 months vs 9.6 months (*P* = .002).^24^ However, the small sample size (149 and 159 patients in the GnP and FFX groups) and the single‐center nature of the study limit its generalizability to the population level.^24^

While multiple real‐world studies have included uLAPC patients in addition to mPC patients, many reporting no significant OS differences between FFX and GnP, all of these studies are limited by small sample sizes and do not compare treatment outcomes by subgroup (mPC and uLAPC).^25^, ^26^, ^27^, ^28^ Within our uLAPC subcohort, OS was significantly greater for FFX compared to GnP and medians were greater for uLAPC patients than mPC patients by 5.2 months for FFX and 2.0 months for GnP. While rates of ED visits were significantly lower and rates of hospitalization for febrile neutropenia were significantly higher in mPC patients receiving FFX compared to GnP, no significant differences for either of these were found for uLAPC patients. Risk of all‐cause hospitalizations remained lower for FFX patients in both mPC and uLAPC subcohorts.

At the time of this study, laboratory data were not available in Ontario, thus we are unable to comment on the effect of missing covariates such as baseline carbohydrate antigen 19‐9 (CA19‐9) and neutrophil‐lymphocyte ratio (NLR), high levels of which have both been significantly correlated with worse OS.^5^, ^24^, ^29^ However, while being a population‐based study we were able to include ECOG PS, an important potential confounder, as we prospectively collected ECOG PS at baseline enrollment for all patients in Ontario as part of the Cancer Care Ontario Real‐World Evidence Initiatives. There may also exist other selection factors associated with the choice of treatment that are not measured, and thus cannot be balanced. That is, confounding by indication may have influenced the observed treatment effects.

In the real world, implementation of universal public funding of FFX for mPC was associated with improved OS compared to patients treated with GnP. In addition, patients treated with FFX had less frequent all‐cause ED visits and all‐cause hospitalization but increased febrile neutropenia‐related hospitalization. Expanding funding of FFX to include uLAPC was associated with a similar trend in benefits, but with improved absolute survival.

## CONFLICT OF INTEREST

Brandon Meyers: Speakers bureau—Celgene.

## AUTHOR CONTRIBUTIONS

Kelvin K. W. Chan, Sierra Cheng, Ruby Redmond‐Misner, Jaclyn Marie Beca, Wanrudee Isaranuwatchai, Lucy Qiao, Craig Earle, Scott R. Berry, James Joseph Biagi, Stephen Welch, Brandon M. Meyers, Nicole Mittmann, Natalie Coburn, Jessica Arias, Deborah Schwartz, Wei Fang Dai, Scott Gavura, Robin McLeod, and Erin Diane Kennedy involved in data interpretation, reviewing, and editing of the manuscript. Kelvin K. W. Chan, Helen Guo, and Lucy Qiao also involved in methodology. Sierra Cheng and Helen Guo also performed writing of the original draft. Helen Guo and Lucy Qiao also involved in formal analysis.
