# Supplementary material for: Real‐world outcomes of FOLFIRINOX vs gemcitabine and nab‐paclitaxel in advanced pancreatic cancer: A population‐based propensity score‐weighted analysis
Source: Cancer Med. 2019 Nov 13;9(1):160–9. doi: 10.1002/cam4.2705 (PMC6943167; doi:10.1002/cam4.2705)
Supplement: Supplementary file 1 [file CAM4-9-160-s001.docx]

Appendix

**Data sources**

Appendix Figure 1 show the data sources used to identify the study population.

New Drug Funding Program Database (NDFP): Contains demographic and detailed cancer drug and disease indication information for patients receiving publicly-funded, newer and often very expensive, injectable cancer drugs administered in hospitals and cancer centres. We used this database to identify patients who received FFX or GnP as first-line treatment.

Cancer Activity Level Reporting (ALR) database: Includes patient-level activity focused on radiation and systemic therapy services. The database was used to obtain previous adjuvant systemic and radiation treatments.

Discharge Abstracts Database (DAD): Contains demographic, administrative and clinical data for hospital discharges; the database was used to obtain previous pancreatic resection, calculate the Charlson comorbidity index and identify hospitalization.

The National Ambulatory Care Reporting System (NACRS): Contains data for all hospital-based and community-based ambulatory care; the database was used to obtain patients’ emergency department visits.

The Registered Persons Database (RPDB): Contains information on persons registered under the Ontario Health Insurance Plan and who are eligible for the Ontario Drug Program; the database was used to obtain patients’ vital statistics.

Ontario Cancer Registry (OCR): A computerized database of information about all Ontario residents who have been diagnosed of invasive neoplasia, except for basal cell and squamous cell skin cancers.

The above data linkages were done through patients’ unique health card numbers. Canadian 2016 Census data was used to obtain patients’ rural/urban status and neighbourhood income quintiles based on postal code of patients’ residence.

**Appendix Figure 1. Data Sources**


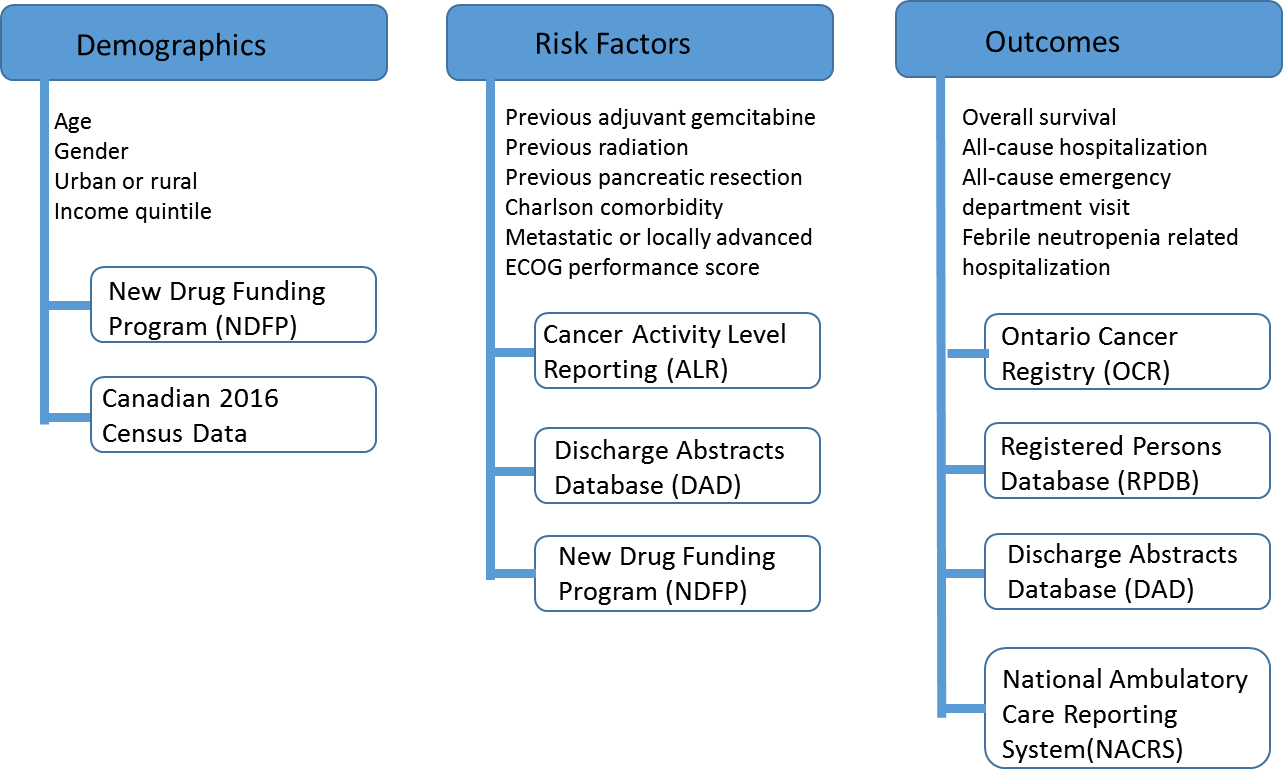


**Appendix Figure 2. Flowchart**


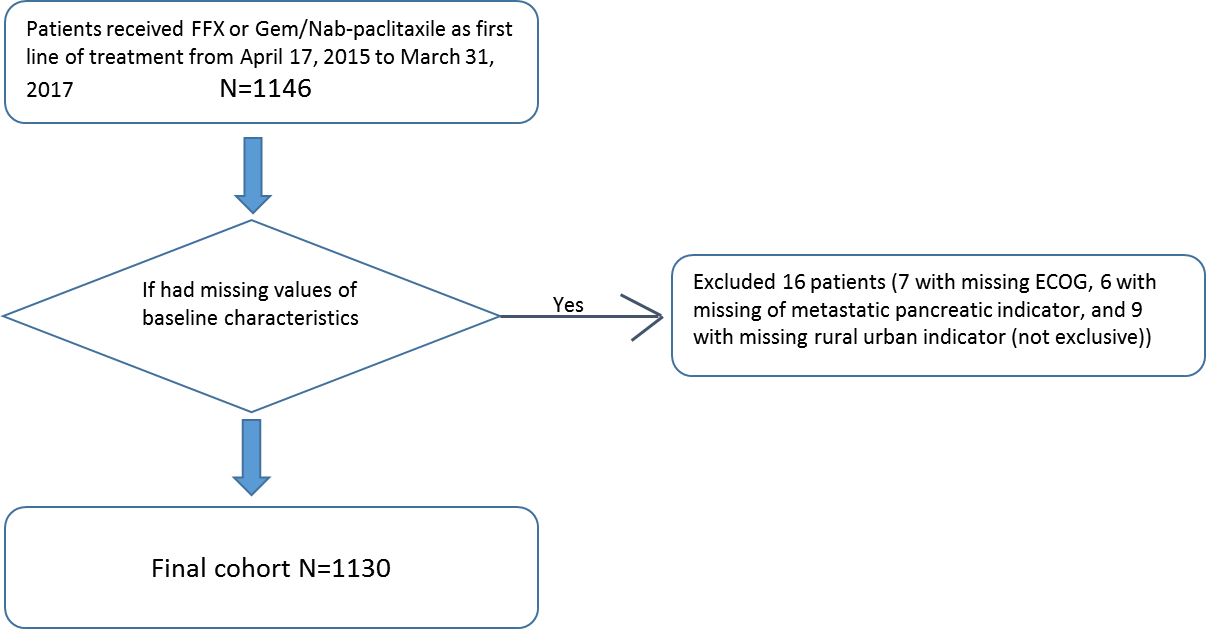


**Appendix Table 1. Baseline characteristics for patients with metastatic pancreatic cancer by chemotherapy treatment**

|  | **Before IPTW** | | | **After IPTW** | | |
| --- | --- | --- | --- | --- | --- | --- |
| **Characteristics*** | **FOLFIRINOX** | **Gemcitabine/ nab-paclitaxel** | **P-value** | **FOLFIRINOX** | **Gemcitabine/ nab-paclitaxel** | **Weighted standardized difference** |
|  | **(n=416)** | **(n=380)** |  | **(n=416)** | **(n=380)** |  |
| **Age at first treatment (mean±sd)** | 61.34 ± 9.33 | 69.07 ± 8.71 | <.0001 | 64.61±12.79 | 64.54±15.01 | 0.0048 |
| **Female** | 197 (47.36%) | 151 (39.74%) | 0.0304 | 42.89% | 43.33% | 0.0090 |
| **Previous adjuvant gemcitabine** | 69 (16.59%) | 34 (8.95%) | 0.0013 | 13.02% | 11.21% | 0.0554 |
| **Previous radiation** | 33 (7.93%) | 40 (10.53%) | 0.2054 | 9.62% | 10.43% | 0.0269 |
| **Previous pancreatic resection** | 86 (20.67%) | 58 (15.26%) | 0.0476 | 18.50% | 18.66% | 0.0043 |
| **Charlson comorbidity index 1+** | 125 (30.05%) | 124 (32.63%) | 0.4323 | 30.46% | 30.89% | 0.0093 |
| **ECOG PS 1+** | 251 (60.34%) | 311 (81.84%) | <.0001 | 70.92% | 70.47% | 0.0100 |
| **Urban** | 361 (86.78%) | 337 (88.68%) | 0.4138 | 12.47% | 12.01% | 0.0141 |
| **Income quintile** |  |  |  |  |  |  |
| **1 (lowest)** | 49 (11.78%) | 66 (17.37%) | 0.2359 | 14.18% | 14.62% | 0.0125 |
| **2** | 75 (18.03%) | 67 (17.63%) |  | 18.93% | 16.54% | 0.0626 |
| **3** | 70 (16.83%) | 69 (18.16%) |  | 16.90% | 16.60% | 0.0079 |
| **4** | 84 (20.19%) | 74 (19.47%) |  | 20.76% | 22.09% | 0.0325 |
| **5 (highest)** | 94 (22.60%) | 72 (18.95%) |  | 20.48% | 21.83% | 0.0330 |
| **unknown** | 44 (10.58%) | 32 (8.42%) |  | 8.76% | 8.32% | 0.0156 |

*Percentage shown in the table is column %
IPTW = Inverse probability treatment weighting
sd = Standard deviation
ECOG PS = Eastern Co-operative Oncology Group performance status

**Appendix Table 2. Baseline characteristics for patients with locally advanced unresectable pancreatic cancer by chemotherapy treatment**

|  | **Before IPTW** | | | **After IPTW** | | |
| --- | --- | --- | --- | --- | --- | --- |
| **Characteristics*** | **FOLFIRINOX** | **Gemcitabine/ nab-paclitaxel** | **P-value** | **FOLFIRINOX** | **Gemcitabine/ nab-paclitaxel** | **Weighted standardized difference** |
|  | **(n=216)** | **(n=118)** |  | **(n=216)** | **(n=118)** |  |
| **Age at first treatment (mean±sd)** | 62.76 ± 8.64 | 69.38 ± 8.64 | <.0001 | 64.65±10.49 | 63.49±17.72 | 0.0795 |
| **Female** | 90 (41.67%) | 47 (39.83%) | 0.7443 | 40.70% | 41.70% | 0.0203 |
| **Previous adjuvant gemcitabine** | 9 (4.17%) | NA | 0.0889 | 3.08% | 3.83% | 0.0409 |
| **Previous radiation** | 8 (3.70%) | 12 (10.17%) | 0.0173 | 6.71% | 5.05% | 0.0707 |
| **Previous pancreatic resection** | 16 (7.41%) | 6 (5.08%) | 0.4134 | 6.67% | 8.97% | 0.0858 |
| **Charlson comorbidity index 1+** | 58 (26.85%) | 38 (32.20%) | 0.3016 | 28.12% | 30.25% | 0.0469 |
| **ECOG PS 1+** | 126 (58.33%) | 96 (81.36%) | <.0001 | 65.88% | 62.95% | 0.0611 |
| **Urban** | 187 (86.57%) | 102 (86.44%) | 0.9728 | 15.22% | 13.01% | 0.0636 |
| **Income quintile** |  |  |  |  |  |  |
| **1 (lowest)** | 31 (14.35%) | 19 (16.10%) | 0.9354 | 15.29% | 20.22% | 0.1294 |
| **2** | 27 (12.50%) | 15 (12.71%) |  | 12.44% | 10.19% | 0.0712 |
| **3** | 41 (18.98%) | 27 (22.88%) |  | 19.81% | 17.55% | 0.0579 |
| **4** | 47 (21.76%) | 24 (20.34%) |  | 21.74% | 18.38% | 0.0839 |
| **5 (highest)** | 43 (19.91%) | 20 (16.95%) |  | 19.27% | 23.13% | 0.0946 |
| **unknown** | 27 (12.50%) | 13 (11.02%) |  | 11.46% | 10.53% | 0.0296 |

*Percentage shown in the table is column %
IPTW = Inverse probability treatment weighting
sd = Standard deviation
ECOG PS = Eastern Co-operative Oncology Group performance status

NA: data not shown because of small cell policy for count less than 6

**Appendix Table 3. Rate ratio (95% CI) of toxicity outcomes for FOLFIRINOX versus Gemcitabine + nab-paclitaxel**

| **Patients** | **Outcome** | **Crude negative binomial model** | **Weighted negative binomial model** | **Adjusted negative binomial model** |
| --- | --- | --- | --- | --- |
| **All pancreatic cancer** | **All-cause ED visit** | 0.72 (0.62, 0.84)  P<.0001 | 0.78 (0.68, 0.91)  P=0.0012 | 0.78 (0.66, 0.93)  P=0.0053 |
|  | **All-cause Hospitalization** | 0.64 (0.52, 0.77)  P<.0001 | 0.71 (0.59, 0.85)  P=0.0002 | 0.72 (0.58, 0.89)  P=0.0025 |
|  | **Hospitalization for febrile neutropenia*** | 1.85 (1.33, 2.57)  P=0.0003 | 2.65 (1.90, 3.68)  P<.0001 | 1.75 (1.23, 2.50)  P=0.0021 |
| **Metastatic pancreatic cancer** | **All-cause ED visit** | 0.75 (0.63, 0.90)  P=0.0025 | 0.80 (0.67, 0.96)  P=0.0151 | 0.78 (0.64, 0.96)  P=0.0194 |
|  | **All-cause Hospitalization** | 0.67 (0.53, 0.85)  P=0.0009 | 0.73 (0.58, 0.91)  P=0.0054 | 0.72 (0.56, 0.94)  P=0.0149 |
|  | **Hospitalization for febrile neutropenia** | 1.53 (0.55, 4.24)  P=0.4100 | 1.97 (0.72, 5.38)  P=0.1871 | 1.83 (0.52, 6.37)  P=0.3444 |
| **Unresectable locally advanced pancreatic cancer** | **All-cause ED visit** | 0.67 (0.51, 0.88)  P=0.0044 | 0.76 (0.59, 0.99)  P=0.0397 | 0.78 (0.57, 1.06)  P=0.1082 |
|  | **All-cause Hospitalization** | 0.60 (0.42, 0.84)  P=0.0028 | 0.68 (0.49, 0.94)  P=0.0178 | 0.71 (0.49, 1.02)  P=0.0662 |
|  | **Hospitalization for febrile neutropenia*** | 2.47 (1.19, 5.13)  P=0.0150 | 3.09 (1.64, 5.84)  P=0.0005 | 1.31 (0.63, 2.72)  P=0.4761 |

*Estimated from Poisson model
ED = Emergency department

**Appendix Table 4. Sensitivity analysis by adjusted income quintile in IPTW model for uLAPC**

| **Outcome** | **IPTW** | | **IPTW + income quintile** | |
| --- | --- | --- | --- | --- |
|  | **HR (95% CI)** | **P-value** | **HR (95% CI)** | **P-value** |
| **Overall mortality** | 0.57 (0.47, 0.70) | <.0001 | 0.55 (0.45, 0.66) | <.0001 |
|  | **OR (95% CI)** | **P-value** | **OR (95% CI)** | **P-value** |
| **All-cause ED visit** | 0.90 (0.63, 1.29) | 0.5772 | 0.89 (0.62, 1.28) | 0.5273 |
| **All-cause Hospitalization** | 0.62 (0.45, 0.85) | 0.0032 | 0.59 (0.43, 0.82) | 0.0017 |
| **Hospitalization for febrile neutropenia** | 1.78 (0.85, 3.74) | 0.1273 | 1.80 (0.85, 3.80) | 0.1249 |
|  | **RR (95% CI)** | **P-value** | **RR (95% CI)** | **P-value** |
| **All-cause ED visit** | 0.76 (0.59, 0.99) | 0.0397 | 0.77 (0.59, 0.99) | 0.0414 |
| **All-cause Hospitalization** | 0.68 (0.49, 0.94) | 0.0178 | 0.69 (0.50, 0.96) | 0.0256 |
| **Hospitalization for febrile neutropenia*** | 3.09 (1.64, 5.84) | 0.0005 | 2.96 (1.59, 5.5) | 0.0006 |

*Estimated from Poisson model
uLAPC = unresectable locally advanced pancreatic cancer
IPTW = Inverse probability treatment weighting
HR = Hazard ratio
OR = Odds ratio
RR = Rate ratio
